# Eutectic Formation of Naproxen with Some Dicarboxylic Acids

**DOI:** 10.3390/pharmaceutics13122081

**Published:** 2021-12-04

**Authors:** Dahye Kim, Soeun Jang, Il Won Kim

**Affiliations:** Department of Chemical Engineering, Soongsil University, Seoul 06978, Korea; byeomilkong@soongsil.ac.kr (D.K.); linda715@soongsil.ac.kr (S.J.)

**Keywords:** active pharmaceutical ingredient, naproxen, dicarboxylic acid, eutectic, dissolution

## Abstract

Eutectic formation with additives is one of the established methods to improve the dissolution behaviors of active pharmaceutic ingredients (APIs). The improvement is mainly due to the increase in the surface area for dissolution, which originates from the finely divided micro-domains generated through the phase separation of the miscible liquid components upon solidification. The present study is to identify eutectic-forming additives for naproxen (NPX), a class II API of the biopharmaceutical classification system. A particular aim was to develop a eutectic mixture with NPX at least over 20 wt%, a minimum to be practical for oral delivery. Screening based on the proximity of the solubility parameter values identified dicarboxylic acids (succinic acid, glutaric acid, and suberic acid) as desirable additives for NPX. Binary melting diagrams were constructed to confirm the eutectic compositions, and the eutectic mixture with suberic acid (NPX 55 wt%) was further investigated. The dissolution (at pH 5.0) of the melt crystallized eutectics was enhanced compared to the simple physical mixture of the same compositions and neat NPX, which was attributed to the microscopically observed lamellar structures. The current study should support the systematic investigations of API eutectic mixtures by selecting appropriate eutectic-forming additives.

## 1. Introduction

Inadequate dissolution behavior of active pharmaceutical ingredients (APIs) is a recurring problem, hindering the development of their oral dosage forms [1,2]. These APIs are specifically categorized as class II or IV in the biopharmaceutical classification system (BCS), depending on their permeability. As such, diverse methods have been devised to alleviate the dissolution problem [3]. The approaches are largely grouped into the control of solubility and surface area, both of which are the key factors proportional to the dissolution rate, as described in the classic Noyes–Whitney equation [4]. The approach to alter the solubility of the APIs includes the formation of suitable salts, polymorphs, cocrystals, amorphous phases, and so on [5,6,7,8]. The process to increase the surface area in contact with the dissolving fluids is micronization and nanonization, for which various top-down (e.g., milling and high-pressure homogenization) and bottom-up (e.g., phase separation and precipitation) processes are in use [6,8,9,10,11].

Eutectic formation is one of the phase separation methods used to spontaneously generate the API domains of reduced size [12,13,14,15]. Traditional methods to form eutectic mixtures have utilized hydrophilic additives, which are readily soluble, as in the pairs of sulfathiazole/urea and griseofulvin/succinic acid [16,17,18]. The resulting structure has the API microcrystals dispersed with the additive domains, and the subsequent dissolution of the additives results in the fine API crystals with a large surface area [12,14,19,20]. Additionally, relatively hydrophobic additives have recently proved to be quite effective once a eutectic structure has been established, and cocrystals (rather than neat APIs) can be the components of the eutectic mixtures [21,22,23,24].

While the eutectic formation has (when possible) proved advantageous in improving API dissolution behaviors, it is not always a straightforward task to predict the feasibility of the API/additive pairs before an experimental endeavor. Still, there are theoretical approaches which allow qualitative screening of potential additive candidates. One of the simplest prediction methods is to use the difference of solubility parameters (δ) between the compound pairs to evaluate their miscibility when liquefied [25,26]. The main advantage is that the δ values can be calculated solely based on the molecular structures of the compounds using either group contribution methods or using a molecular dynamics simulation [25,26,27,28,29]. Another noncomplex proposal for the prediction was using an index (*I_c_*) based on the van ’t Hoff equation:

(1) $$I_{c}=\frac{\Delta H_{fus,API}}{R}\;\frac{T_{fus,API}-T_{fus,add}}{{\left(T_{fus,API}\right)}^{2}}$$

where Δ*H_fus,API_* and *T_fus,API_* are the molar enthalpy of fusion and melting point of an API; *T_fus,add_* is the melting point of an additive; *R* is gas constant [13]. It utilizes thermodynamic parameters, such as melting points and the enthalpy of fusion, to calculate the initial rate of melting point depression, with respect to the melting point difference between the compounds. Therefore, a logical and synergistic procedure would involve first screening the probable API/additive pairs, based on Δδ, and then analyzing experimentally determined melting diagrams with *I_c_*.

**Figure 1:**
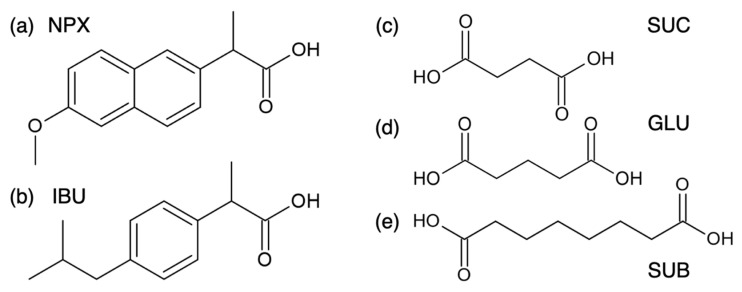
Molecular structures of (**a**) naproxen (NPX), (**b**) ibuprofen (IBU), (**c**) succinic acid (SUC), (**d**) glutaric acid (GLU), and (**e**) suberic acid (SUB).

In the present study, naproxen (NPX) was combined with some dicarboxylic acids (Figure 1), which were screened based on the Δδ and *I_c_*. Ibuprofen (IBU) was also tested with the dicarboxylic acids for comparison. We have previously reported unsuccessful NPX eutectic formation with some fatty alcohols, with which similarly structured IBU successfully formed eutectics [21]. In addition, a particular aim was to obtain an NPX eutectic mixture with the API over 20 wt%. The cases of naproxen eutectic formation have been scarcely reported (especially with small molecules), and the maximum NPX content was only about 15 wt%, making the eutectic compositions impractical by limiting the NPX dose to less than 150 mg, if the upper limit of the mixture is assumed to be 1000 mg [13,26,30,31].

## 2. Materials and Methods

### 2.1. Materials

Two active pharmaceutical ingredients (APIs) and three dicarboxylic acids were purchased from Sigma-Aldrich (St. Louis, MO, USA): S-naproxen (NPX, C_14_H_14_O_3_: ≥ 98.5%, USP testing specifications), ibuprofen (IBU, C_13_H_18_O_2_: ≥ 98%), succinic acid (SUC, C_4_H_6_O_4_: ≥ 99%), glutaric acid (GLU, C_5_H_8_O_4_: 99%), and suberic acid (SUB, C_8_H_14_O_4_: 98%).

Chemicals for dissolution media were as follows: FaSSIF/FeSSIF/FaSSGF powder (Biorelevant, London, UK), sodium chloride (NaCl: ≥ 99.5%, Sigma-Aldrich), sodium hydroxide (NaOH: ≥ 97%, Sigma-Aldrich), acetic acid (CH_3_COOH: ≥ 99%, Sigma-Aldrich), 1 M HCl (aq) (Samchun Chemical, Seoul, Korea), and 1 M NaOH (aq) (Daejung Chemical, Gyeonggi, Korea). Additionally, deionized (DI) water (resistivity > 18.2 MΩ·cm) was obtained from a Direct-Q3 water purification system (Millipore, Burlington, MA, USA).

### 2.2. Thermal Analysis for Melting Diagrams

Fine powders of APIs (NPX and IBU) and dicarboxylic acids (SUC, GLU, and SUB) were individually prepared by grinding each compound with an agate mortar and pestle for 2 min. Then, the binary mixtures of each API and dicarboxylic acids were prepared at various molar ratios by mixing them using a mortar and pestle for an additional 2 min.

The mixtures (2–3 mg in a 40 μL Al pan with a pinhole-punched lid) were analyzed using a differential scanning calorimeter (DSC: DSC3 STARe system, Mettler Toledo, Columbus, OH, USA) under a nitrogen atmosphere with the heating rates 1 or 10 °C/min. The instrument was pre-calibrated for temperature and enthalpy using indium and zinc. When the binary mixtures showed eutectic behavior, the values of eutectic melting enthalpy at various compositions were calculated after peak deconvolution using the Peak Analyzer function of Origin 2021 (OriginLab, Northampton, MA, USA).

### 2.3. Characterization of Melt Crystallized NPX/SUB Mixtures

Some NPX/SUB mixtures were melt crystallized for the main purpose of studying their in vitro dissolution behaviors. The mixtures were completely melted in Al dishes by heating the room temperature samples to 25 °C above their liquidus temperatures and maintaining those temperatures for 1–2 min on a hot plate (AREX-6, VELP Scientifica, Usmate Velate, Italy). Then, the liquid was crystallized by placing it in a 25 °C incubator for 24 h (BF-150LI incubator, BioFree, Seoul, Korea).

Microstructures of the melt crystallized samples were investigated using scanning electron microscopy (SEM: GeminiSEM 300, Carl Zeiss, Jena, Germany). The samples were placed on Al stubs using carbon tape and coated with Pt using a sputter coater (Q150R S, Quorum Technologies, Laughton, UK) to minimize charging. The air-side of the melt crystallized samples was observed with the accelerating voltage of 2 kV.

The phase identity of the melt crystallized mixtures was analyzed by X-ray diffraction (XRD). Powder samples were prepared with an agate mortar and pestle (1–2 min grinding), and they were placed on a zero-background sample holder (Bruker AXS, Billerica, MA, USA). The diffraction data (2θ range 5–35° at 0.02° increment, scanning rate 1 °/min) were collected in the θ-θ mode using a D2 PHASER diffractometer (Bruker AXS) with CuK_α_ radiation (λ = 1.5406 Å) at 30 kV and 10 mA.

In vitro release behaviors of NPX (37 °C) were studied using a USP type II apparatus (paddle) at 100 rpm (RC-3 dissolution tester, Minhua Pharmaceutical Machinery, Shanghai, China). Powder samples between sizes of 45 and 300 μm were used by collecting the ground samples between appropriate sieves (Daihan Scientific, Gangwon, Korea). Dissolution media were FeSSIF (pH 5.0) and a pH 5.0 buffer solution. FeSSIF was prepared as instructed by the supplier of FaSSIF/FeSSIF/FaSSGF powder (Biorelevant, London, UK), and it contained sodium taurocholate (15 mM), lecithin (3.75 mM), sodium chloride (203 mM), sodium hydroxide (101 mM), and acetic acid (144 mM). The pH 5.0 buffer solution was the same, except for the absence of sodium taurocholate and lecithin. The amount of NPX in 500 mL media was 250 and 120 mg for FeSSIF and the buffer, respectively (non-sink conditions). Dissolution was monitored at 5, 10, 15, 20, 30, 40, 60, 90, and 120 min by withdrawing 3 mL aliquots of the solution. A constant volume was maintained by immediately adding an equal amount of the fresh solution. UV absorbance (V730, Jasco, Tokyo, Japan) at 331.8 nm (FeSSIF) or 272 nm (pH 5.0 buffer) was measured for the NPX concentration of the aliquots after filtration (0.20 μm PTFE filter, Advantec, Tokyo, Japan). All dissolution experiments were independently repeated in triplicate. The statistical significance of the area under the curve (AUC) was examined via two-sample t-tests using the Statistics function of Origin 2021.

## 3. Results and Discussion

### 3.1. Melting Behaviors of APIs with Dicarboxylic Acids

**Figure 2:**
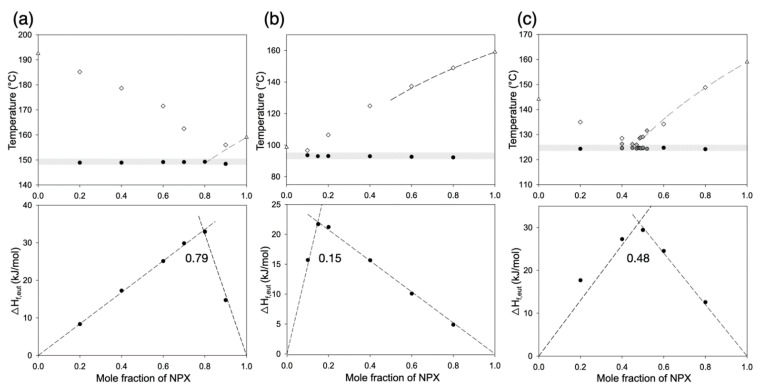
Melting diagrams and Tammann plots of (**a**) NPX/SUC, (**b**) NPX/GLU, and (**c**) NPX/SUB mixtures. Dashed lines in melting diagrams represent the ideal behaviors calculated using the van ’t Hoff equation. A heating rate of 10 °C/min was used for the overall construction of the diagrams (empty triangles for the melting points of pure components; empty diamonds liquidus temperatures; filled black circles solidus temperatures). A heating rate of 1 °C/min (gray diamonds and dark gray circles for the liquidus and solidus temperatures, respectively) was used to determine the eutectic composition of NPX/SUB more accurately as 0.48:0.52. In Tammann plots, eutectic compositions (mole fraction of NPX = 0.79, 0.15, and 0.48, respectively) were predicted as the intersections of the extrapolated lines of the enthalpy of eutectic melting (ΔH_f,eut_), obtained from the deconvolution of the DSC thermograms (heating rate 10 °C/min).

Melting diagrams of API/dicarboxylic acid pairs were experimentally determined using DSC. Figure 2 and Figure S1 show the cases of NPX and IBU, respectively. DSC thermograms for the construction of the diagrams are shown in Figures S2 and S3. The solidus temperatures were determined from the onsets of the first melting, whereas the liquidus temperatures were measured as the vertices of the final endothermic peaks due to the frequently broad nature of the peaks. The choice of the dicarboxylic acids was based on the closeness of their solubility parameters (δ) to that of NPX, as stated in Section 1. Details of the analysis on Δδ, as well as *I_c_*_,_ are presented in Section 3.2.

NPX showed eutectic compositions with all three dicarboxylic acids (SUC, GLU, and SUB) (Figure 2). When the eutectic compositions were estimated using Tammann plots (Figure 2), they were NPX/SUC = 7.9:2.1, NPX/GLU = 1.5: 8.5, and NPX/SUB = 4.8:5.2. The estimated eutectic composition of NPX/SUB was experimentally verified using a slower heating rate (1 °C/min) of the additional DSC scans (gray symbols in Figure 2c). The eutectic composition of NPX/SUB contained a large enough amount of NPX (48 mol%; 55 wt%) to be useful for its oral delivery. For example, a 200 mg NPX tablet would contain ca. 164 mg SUB, making the combined mass of 364 mg well below the usual upper limit (1000 mg) [26,30].

Note that similar time-consuming (1 °C/min) verification for the exact composition was not performed for the NPX/GLU and NPX/SUC, because their eutectic mixtures were of relatively less value due to the following reasons. First, the NPX content of the NPX/GLU eutectic composition (15 mol%; 24 wt%) was on the smaller side and not greatly exceeding those values reported previously (≤15 wt%) [13,26,31]. A 200 mg NPX tablet would contain ca. 650 mg GLU, making the total mass already too close to 1000 mg, even without considering the other potentially necessary excipients [26,30,32]. Secondly, SUC, even with a high NPX content (79 mol%) at the NPX/SUC eutectic point, suffers the anhydride formation and partial sublimation during prolonged heating, making it undesirable for the process of melt recrystallization [33]. Additionally, noted in the NPX/dicarboxylic acid system is the minimal deviation of the melting point depression from the van ’t Hoff equation (dashed lines in Figure 2), which indicates the lack of particularly strong intermolecular interactions between NPX and the dicarboxylic acids [21,23].

IBU formed a eutectic composition only with GLU; no eutectic points were observed with SUC and SUB (Figure S1). The eutectic composition of IBU/GLU was estimated as 0.65:0.35 using a Tammann plot, of which an exact value was not pursued with additional DSC experiments because NPX eutectic was the main focus of the current study. The main purpose of examining the IBU/dicarboxylic acid pairs was to evaluate the predictive power of Δδ and *I_c_*. Detailed analysis on this point appears in Section 3.2.

Overall, an NPX/SUB eutectic composition with about 48 mol% (55 wt%) NPX was identified, which satisfied our initial aim to discover a eutectic point potentially useful for oral delivery and exceed the reported values in literature (≤15 wt%) [13,26,31]. This mixture was further processed using melt recrystallization and characterized for its microstructure and dissolution behavior (Section 3.3). Note that the melt crystallization did not alter the phase identity or crystallinity of the samples as evaluated by XRD and DSC, respectively (Figure S4). This is also in accordance with the successful prediction of the eutectic composition by the Tammann plot (Figure 2), with the inclusion of the intercepts at NPX mole fraction = 0 and 1 (assuming no formation of solid solutions [23,34]). In addition, XRD and DSC data showed virtually no change over the 3 month storage at room temperature (Figure S5).

### 3.2. Screening Molecular Pairs for Successful Eutectic Formation

The initial screening of the additives for NPX was based on the differences in their solubility parameter values (Δδ). Table 1 shows the values of the solubility parameters (δ) for NPX (23.41), IBU (20.95), the dicarboxylic acids (23.50–27.02), and the fatty alcohols (18.44–18.87) calculated using a group contribution method [27]. The structural difference between NPX and IBU is twofold: (i) methoxy of NPX vs. isobutyl of IBU; (ii) naphthyl of NPX vs. phenyl of IBU. The hypothetical change of phenyl of IBU to naphthyl increases the δ value to 21.91, which also can be interpreted as the decrease from 23.37 of NPX by methoxy-to-isobutyl substitution. Therefore, the hydrogen bonding-related methoxy group seems to have a slightly bigger contribution to the increase in the δ value than the π-π stacking-related naphthyl group.

The dicarboxylic acids were a logical choice for the eutectic formation with NPX when the previous attempt with fatty alcohols was not successful [21]. The existence of carboxylic acid groups increased the values of solubility parameters for the dicarboxylic acids making them better candidates for NPX [27]. Especially, the δ value of SUB was nearly identical to that of NPX, which indicates that exceptional compatibility between the two compounds is highly probable.

The order of Δδ (between APIs and dicarboxylic acids) was NPX/SUB << NPX/GLU < IBU/SUB < NPX/SUC < IBU/GLU < IBU/SUC. The overall prediction on the tendency for the eutectic formation was satisfactory, except that IBU/SUB was incorrectly predicted as one of the preferable pairs. Therefore, the use of Δδ is valid only for the overall trend, and its qualitative nature certainly does not eliminate the necessity of experiments. We also caution here that different calculation methods generating slightly different values cannot be mixed for the δ comparison [25,26,27,28]. Still, the merit of using Δδ is that the chemical structures of the compounds are the only required information, making Δδ suitable for the initial screening.

The order of |*I_c_*| based on thermodynamic parameters, such as melting points and enthalpy, was NPX/SUB < IBU/GLU < NPX/SUC < NPX/GLU < IBU/SUB < IBU/SUC (Table 2; Table S1 for the raw data of the calculations). This sequence was more satisfactory than that of Δδ, because it correctly identified IBU/SUB and IBU/SUC as the least favorable pairs in accordance with the experimental results (Section 3.1). Additionally, shown in Table 2 are the |*I_c_*| of the APIs with the fatty alcohols in the previous study [21]. For the twelve pairs presented in Table 2, the biggest |*I_c_*| among the eutectic forming pairs was 1.28 (NPX/GLU), and the smallest value among the non-eutectic forming ones was 1.80 (NPX/docosanol). Therefore, the critical value appears to be approximately between 1.3 and 1.8 in the systems presented here and in our previous study [21]. Additionally, there appears to exist some correlation between the *I_c_* value and the mole fraction of APIs at eutectic points (Figure S6). However, it remains to be seen if the correlation would extend to the other APIs with completely different molecular structures from IBU and NPX.

The original evaluation method with *I_c_* was developed for poly (ethylene glycol) as a eutectic forming additive, and the critical value for eutectic/monotectic behavior was 2–2.5 [13]. For the small molecule additives, the critical value appears somewhat lower. The discrepancy probably arises from the difference of the extent to which the initial melting point depression predicts the overall shape of the liquid’s lines, since the *I_c_* is based on the case of a very dilute mixture with a very small melting point depression (i.e., the initial rate of depression) [13,35]. Further experimental and theoretical work would be necessary to elucidate the molecular origin of the discrepancy, although it might be related to the inherent differences in molecular size between polymers and small molecules.

Overall, the proximity of the solubility parameters calculated from the molecular structures was a reasonable indicator to initially screen probable pairs for eutectic formation. Additionally, the *I_c_* based on melting points and enthalpy of fusion was in closer agreement with the experimental observations.

### 3.3. Dissolution Behavior and Microstructure of NPX/SUB Eutectics

The effect of eutectic formation on the dissolution of NPX was investigated at pH 5.0 following the recommendation of NPX intake (with food or milk) [36]. Two extreme conditions of the concentration of the bile salt and phospholipid components (none and FeSSIF) were tested to consider the wide biological variation of the biosurfactants among patients [3,24,37,38].

**Figure 3:**
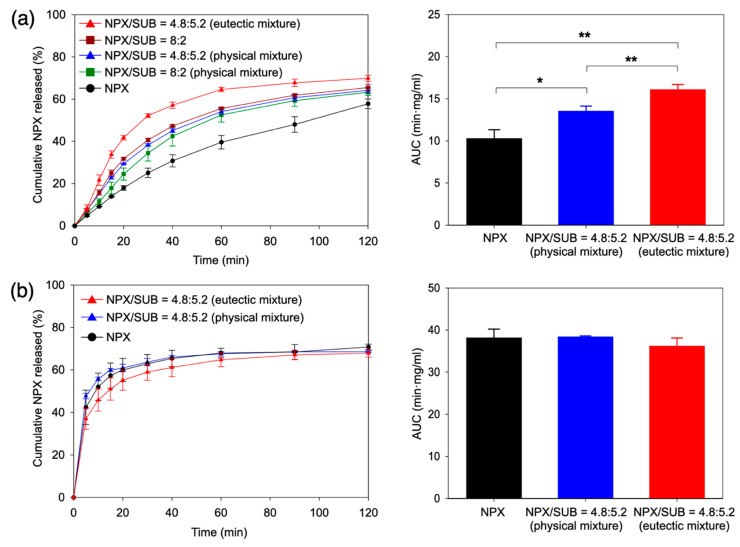
Dissolution profiles of the NPX/SUB eutectic mixture (4.8:5.2) in comparison with the physical mixture and pure NPX: (**a**) pH 5.0 (NPX/SUB = 8:2 was additionally shown); (**b**) FeSSIF (*n* = 3 for both cases). The results of two-sample t-tests for the area under the curve (AUC) are also presented for NPX/SUB = 4.8:5.2 (eutectic and physical mixtures) and neat NPX (* and ** indicate the statistically significant differences with *p* < 0.05 and *p* < 0.01, respectively).

Figure 3a (pH 5.0 without the bile salt and phospholipid addition) shows the enhanced dissolution of the recrystallized eutectic mixture (NPX/SUB = 4.8:5.2), whereas the simple physical mixture at the same composition only showed a marginal increase from neat NPX. The statistically significant differences were measured as the area under the curve (AUC) for the 120 min release. The enhanced release behavior from the eutectic mixture was statistically significant, with *p* < 0.01 against both the physical mixture at the same composition and neat NPX, while the mere existence of SUB offered some degree of enhancement (*p* < 0.05 between the physical mixture and neat NPX).

Additionally, shown in Figure 3a is the case of NPX/SUB = 8:2, where about 25% of NPX forms a eutectic mixture with SUB, the rest existing locally as larger NPX domains. As expected, this partially eutectic composition displayed intermediate release behavior between entirely eutectic mixture (4.8:5.2) and neat NPX. 

Figure 3b displays statistically similar release behavior (*p* > 0.05) among the eutectic mixture, the physical mixture, and neat NPX under the FeSSIF condition. The abundant existence of the biosurfactants acting as the solubilizing agents for NPX appears to overwhelm the differences of the three samples, making their dissolution variation statistically insignificant. 

**Figure 4:**
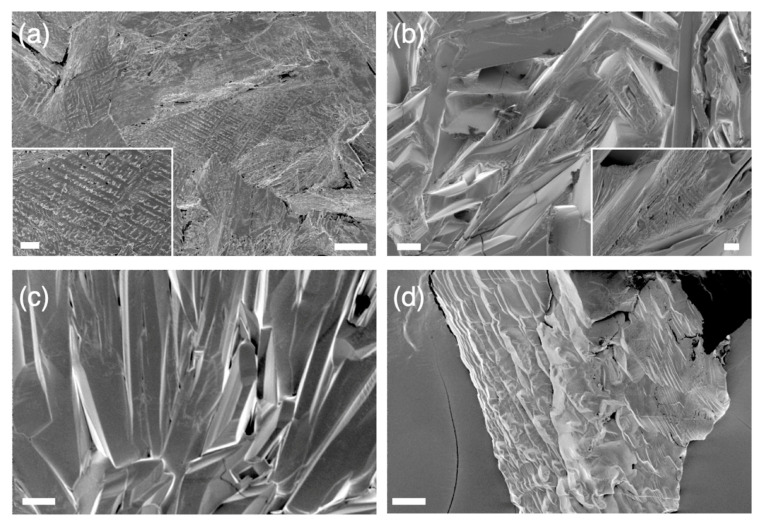
SEM images of NPX/SUB mixtures and neat phases after melt recrystallization: (**a**) 4.8:5.2 (eutectic); (**b**) 8:2; (**c**) neat NPX; (**d**) neat SUB. Scale bars in the main images and insets are 20 μm and 5 μm, respectively.

The origin of the generally enhanced dissolution behavior of the eutectic mixture could be found in its microstructure showing finely divided microcrystals. Figure 4a shows the lamellar microstructures featured in the NPX/SUB = 4.8:5.2, which are typically found in other eutectic mixtures as well [12,39]. The NPX/SUB = 8:2 sample (Figure 4b) showed only partial regions of similarly divided microcrystals. Note that neat NPX (Figure 4c) and neat SUB (Figure 4d) formed bulky crystals, of which feature size was several orders bigger than those found in the eutectics.

Overall, the newly established eutectic composition of NPX/SUB = 4.8:5.2 showed a promising dissolution profile, although the enhanced dissolution was insignificant at the FeSSIF condition with a large amount of bile salt and phospholipid components. Additionally, the finely divided microstructures, which would increase the surface area in contact with liquid, were microscopically observed.

## 4. Conclusions

In summary, we developed a eutectic system between NPX and some dicarboxylic acids (SUC, GLU, and SUB). The choice of eutectic-forming additives specific to NPX was guided by their solubility parameters and experimentally verified along with the pairwise *I_c_* parameters. In particular, the NPX/SUB possessed a eutectic composition at 55 wt% (48 mol%) NPX, exceeding the reported values in the literature (≤15 wt%) and satisfying the requirement to be practical for oral delivery [13,26,30,31]. When the NPX/SUB eutectic mixture was melt recrystallized, it developed a compartmentalized microstructure that significantly enhanced its dissolution behavior in the absence of biosurfactants. The current study, along with our previous investigations on eutectics, should provide systematic information for devising API-specific eutectic systems to improve API dissolution behaviors [21,24].

## Figures and Tables

**Table 1:** Solubility parameter (δ) values of APIs, dicarboxylic acids, and fatty alcohols.

Type	Compound	δ (MPa^1/2^)
API	NPX	23.37
	IBU	20.91
Dicarboxylic acid	SUC	27.02
	GLU	25.79
	SUB	23.50
Fatty alcohol ^1^	TD	18.87
	OD	18.61
	DC	18.44

^1^ Tetradecanol (TD), octadecanol (OD), and docosanol (DC).

**Table 2:** The *I_c_* values for the API/dicarboxylic acid and API/fatty alcohol mixtures.

Type	Compound	*I_c_*	Compound	*I_c_*
API/ Dicarboxylic acid	NPX/SUC	−0.71	IBU/SUC	−3.12
	NPX/GLU	1.28	IBU/GLU	−0.62
	NPX/SUB	0.31	IBU/SUB	−1.83
API/ Fatty alcohol ^1^	NPX/TD	2.52	IBU/TD	0.94
	NPX/OD	2.08	IBU/OD	0.40
	NPX/DC	1.80	IBU/DC	0.04

^1^ Tetradecanol (TD), octadecanol (OD), and docosanol (DC).

## Data Availability

The data presented in this study are contained in this article and its Supplementary Materials.

## Supplementary Materials

The following are available online at https://www.mdpi.com/article/10.3390/pharmaceutics13122081/s1, Table S1. Raw data for the calculation of *I_c_* values; Figure S1: Melting diagrams of (**a**) IBU/SUC, (**b**) IBU/GLU (with the Tammann plot), and (**c**) IBU/SUB mixtures; Figure S2. DSC thermograms of (**a**) NPX/SUC, (**b**) NPX/GLU, and (**c**,**d**) NPX/SUB mixtures. Heating rate 10 °C/min for (**a**,**b**,**c**) and 1 °C/min for (**d**); Figure S3. DSC thermograms of (**a**) IBU/SUC, (**b**) IBU/GLU, and (**c**) IBU/SUB mixtures; Figure S4. (**a**) XRD patterns for melt recrystallized NPX, NPX/SUB, and SUB. (**b**) DSC thermograms to compare NPX and NPX/SUB before and after melt recrystallization; Figure S5. (**a**) XRD patterns and (**b**) DSC thermograms to show that virtually no change occurred for the 3 month storage (room temperature) after melt crystallization of NPX/SUB; Figure S6. Correlation between *I_c_* and the mole fraction of APIs at eutectic points.

## References

[1] Kawabata Y., Wada K., Nakatani M., Yamada S., Onoue S. (2011). Formulation design for poorly water-soluble drugs based on biopharmaceutics classification system: Basic approaches and practical applications. Int. J. Pharm..

[2] Loftsson T., Brewster M.E. (2010). Pharmaceutical applications of cyclodextrins: Basic science and product development. J. Pharm. Pharmacol..

[3] Florence A.T., Attwood D. (2016). Physicochemical Principles of Pharmacy: In Manufature, Formulation and Clinical Use.

[4] Noyes A.A., Whitney W.R. (1897). The rate of solution of solid substances in their own solution. J. Am. Chem. Soc..

[5] He X., Qiu Y., Chen Y., Zhang G.G.Z., Liu L., Porter W.R. (2009). Integration of physical, chemical, mechanical, and biopharmaceutical properties in solid oral dosage form development. Developing Solid Oral Dosage Forms.

[6] Boyd B.J., Bergström C.A.S., Vinarov Z., Kuentz M., Brouwers J., Augustijns P., Brandl M., Bernkop-Schnürch A., Shrestha N., Préat V. (2019). Successful oral delivery of poorly water-soluble drugs both depends on the intraluminal behavior of drugs and of appropriate advanced drug delivery systems. Eur. J. Pharm. Sci..

[7] Jones W., Motherwell W.D.S., Trask A.V. (2006). Pharmaceutical cocrystals: An emerging approach to physical property enhancement. MRS Bull..

[8] Jermain S.V., Brough C., Williams R.O. (2018). Amorphous solid dispersions and nanocrystal technologies for poorly water-soluble drug delivery–An update. Int. J. Pharm..

[9] Kesisoglou F., Panmai S., Wu Y. (2007). Nanosizing–Oral formulation development and biopharmaceutical evaluation. Adv. Drug. Deliv. Rev..

[10] Shegokar R., Müller R.H. (2010). Nanocrystals: Industrially feasible multifunctional formulation technology for poorly soluble actives. Int. J. Pharm..

[11] Choi I., Park S.Y., Lee S.-W., Kang Z., Jin Y.S., Kim I.W. (2020). Dissolution enhancement of sorafenib tosylate by co-milling with tetradecanol post-extracted using supercritical carbon dioxide. Pharmazie.

[12] Cherukuvada S., Nangia A. (2014). Eutectics as improved pharmaceutical materials: Design, properties and characterization. Chem. Commun..

[13] Law D., Wang W., Schmitt E.A., Long M.A. (2002). Prediction of poly(ethylene glycol)-drug eutectic compositions using an index based on the van’t Hoff equation. Pharm. Res..

[14] Law D., Wang W., Schmitt E.A., Qiu Y., Krill S.L., Fort J.J. (2003). Properties of rapidly dissolving eutectic mixtures of poly(ethylene glycol) and fenofibrate: The eutectic microstructure. J. Pharm. Sci..

[15] Vippagunta S.R., Wang Z., Hornung S., Krill S.L. (2007). Factors affecting the formation of eutectic solid dispersions and their dissolution behavior. J. Pharm. Sci..

[16] Sekiguchi K., Obi N. (1961). Studies on absorption of eutectic mixture. I. A comparison of the behavior of eutectic mixture of sulfathiazole and that of ordinary sulfathiazone in man. Chem. Pharm. Bull..

[17] Goldberg A.H., Gibaldi M., Kanig J.L. (1966). Increasing dissolution rates and gastrointestinal absorption of drugs via solid solutions and eutectic mixtures. III. Experimental evaluations of griseofulvin–succinic acid solid solution. J. Pharm. Sci..

[18] Chiou W.L., Niazi S. (1973). Differential thermal analysis and X-ray diffraction studies of griseofulvin-succinic acid solid dispersions. J. Pharm. Sci..

[19] Figueirêdo C.B.M., Nadvorny D., de Medeiros Vieira A.C.Q., Sobrinho J.L.S., Neto P.J.R., Lee P.I., de La Roca Soares M.F. (2017). Enhancement of dissolution rate through eutectic mixture and solid solution of posaconazole and benznidazole. Int. J. Pharm..

[20] Bazzo G.C., Pezzini B.R., Stulzer H.K. (2020). Eutectic mixtures as an approach to enhance solubility, dissolution rate and oral bioavailability of poorly water-soluble drugs. Int. J. Pharm..

[21] Jin S., Jang J., Lee S., Kim I.W. (2020). Binary mixtures of some active pharmaceutical ingredients with fatty alcohols—the criteria of successful eutectic formation and dissolution improvement. Pharmaceutics.

[22] Évora A.O.L., Castro R.A.E., Maria T.M.R., Silva M.R., Ter Horst J.H., Canotilho J., Eusébio M.E.S. (2014). A thermodynamic based approach on the investigation of a diflunisal pharmaceutical co-crystal with improved intrinsic dissolution rate. Int. J. Pharm..

[23] Évora A.O.L., Castro R.A.E., Maria T.M.R., Silva M.R., Ter Horst J.H., Canotilho J., Eusébio M.E.S. (2016). Co-crystals of diflunisal and isomeric pyridinecarboxamides–a thermodynamics and crystal engineering contribution. CrystEngComm.

[24] Kim H., Jang S., Kim I.W. (2021). Enhanced dissolution of naproxen by combining cocrystallization and eutectic formation. Pharmaceutics.

[25] Greenhalgh D.J., Williams A.C., Timmins P., York P. (1999). Solubility parameters as predictors of miscibility in solid dispersions. J. Pharm. Sci..

[26] Dinge A. (2012). Eutectic Mixtures of Drugs with Poor Aqueous Solubility—Solid State Characterization and Dissolution Studies. Ph.D. Thesis.

[27] Fedors R.F. (1974). A method for estimating both the solubility parameters and molar volumes of liquids. Polym. Eng. Sci..

[28] Gupta J., Nunes C., Vyas S., Jonnalagadda S. (2011). Prediction of solubility parameters and miscibility of pharmaceutical compounds by molecular dynamics simulations. J. Phys. Chem. B.

[29] Ponnammal P., Kanaujia P., Yani Y., Ng W.K., Tan R.B.H. (2018). Orally disintegrating tablets containing melt extruded amorphous solid dispersion of tacrolimus for dissolution enhancement. Pharmaceutics.

[30] Leuner C., Dressman J. (2000). Improving drug solubility for oral delivery using solid dispersions. Eur. J. Pharm. Biopharm..

[31] Morgan C. (2014). Investigating Eutectic Mixtures for Poorly Soluble Compounds. Master’s Thesis.

[32] Niazi S.K. (2009). Handbook of Pharmaceutical Manufacturing Formulations: Compressed Solid Products.

[33] Yu Q., Dang L., Black S., Wei H. (2012). Crystallization of the polymorphs of succinic acid via sublimation at different temperatures in the presence or absence of water and isopropanol vapor. J. Cryst. Growth.

[34] Corvis Y., Négrier P., Lazerges M., Massip S., Léger J.-M., Espeau P. (2010). Lidocaine/L-menthol binary system: Cocrystallization versus solid-state immiscibility. J. Phys. Chem. B.

[35] Levine I.N. (2009). Physical Chemistry.

[36] Lacy C.F., Armstrong L.L., Goldman M.P., Lance L.L. (2008). Drug Information Handbook: A Comprehensive Resource for All Clinicians and Healthcare Professionals.

[37] Galia E., Nicolaides E., Hörter D., Löbenberg R., Reppas C., Dressman J.B. (1998). Evaluation of various dissolution media for predicting in vivo performance of class I and II drugs. Pharm. Res..

[38] Lipert M.P., Roy L., Childs S.L., Rodríguez-Hornedo N. (2015). Cocrystal solubilization in biorelevant media and its prediction from drug solubilization. J. Pharm. Sci..

[39] Callister W.D., Rethwisch D.G. (2014). Materials Science and Engineering: An Introduction.

