# Supplementary material for: Eutectic Formation of Naproxen with Some Dicarboxylic Acids
Source: Pharmaceutics. 2021 Dec 4;13(12):2081. doi: 10.3390/pharmaceutics13122081 (PMC8706014; doi:10.3390/pharmaceutics13122081)
Supplement: Supplementary file 1 [file pharmaceutics-13-02081-s001.zip › Supplementary Material/Supplementary materials v3.pdf]

Table S1. Raw data for the calculation of I_c values

Type	Compound	MW (g/mol)	T_{fus} (K)	ΔH_{fus} (J/g)	ΔH_{fus} (kJ/mol)
API	NPX	230.26	432.15	142.55	32.824
	IBU	206.29	348.57	130.35	26.889
Dicarboxylic acid	SUC	-	465.63	-	-
	GLU	-	371.73	-	-
	SUB	-	417.25	-	-
Fatty alcohol [1]	TD	-	313.15	-	-
	OD	-	333.65	-	-
	DC	-	346.98	-	-

[1] Tetradecanol (TD), octadecanol (OD), and docosanol (DC).

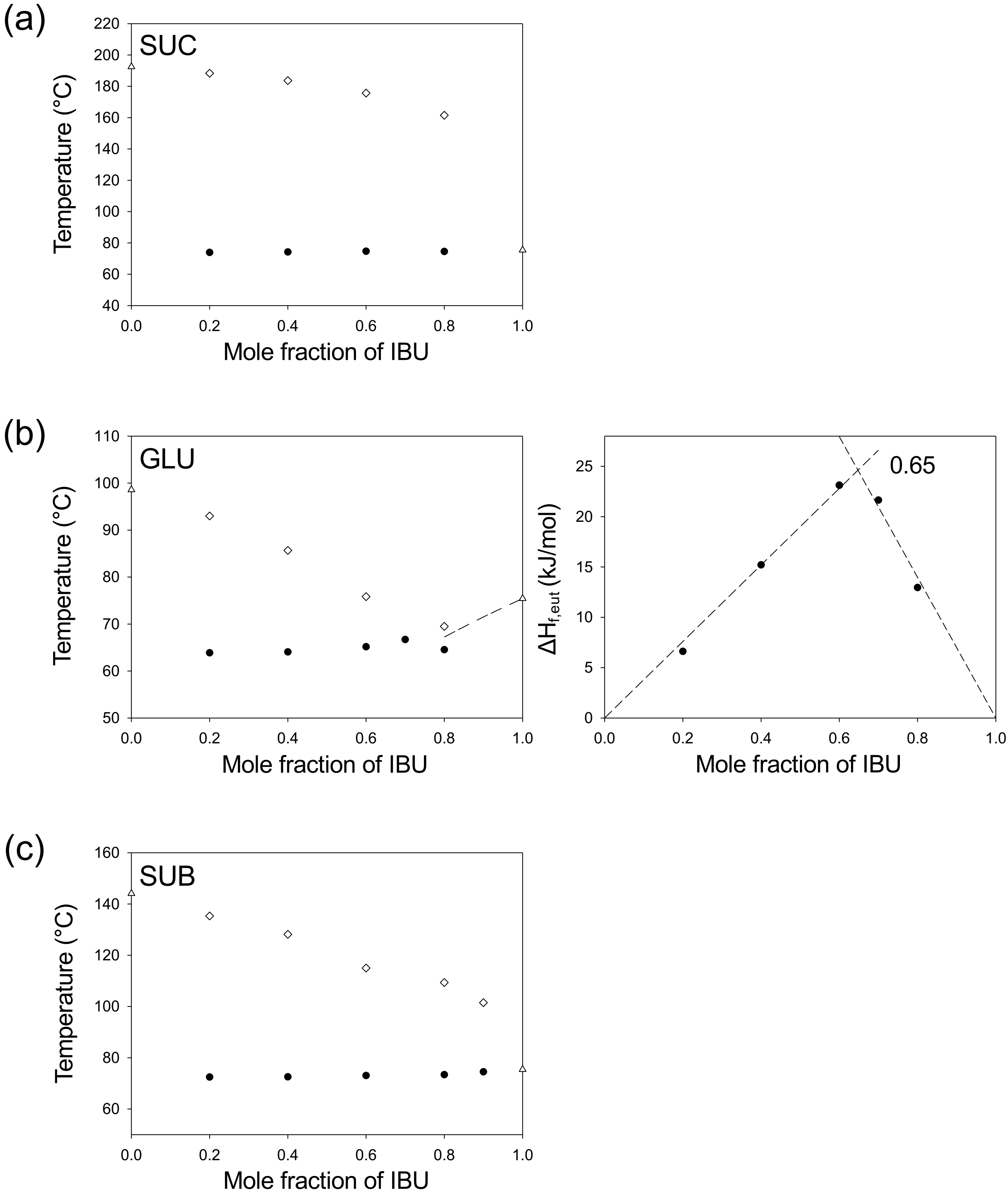

Figure S1. Melting diagrams of (a) IBU/SUC, (b) IBU/GLU (with Tammann plot), and (c) IBU/SUB mixtures.

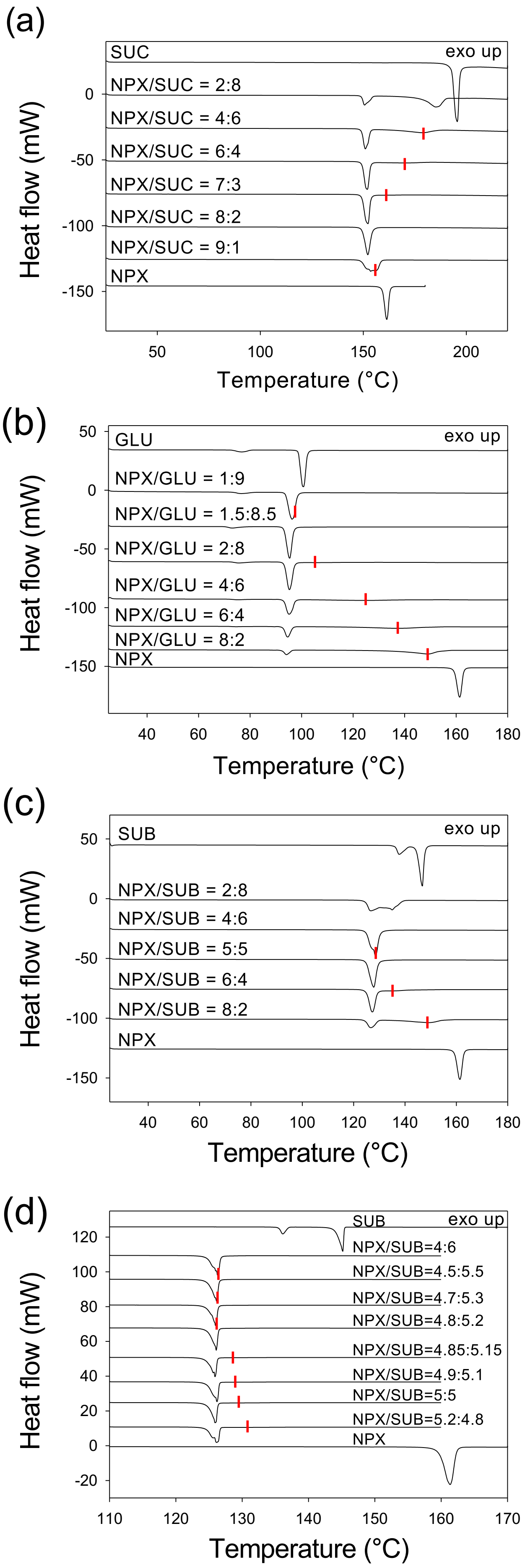

Figure S2. DSC thermograms of (a) NPX/SUC, (b) NPX/GLU, and (c, d) NPX/SUB mixtures. Heating rate 10 °C/min for (a, b, c) and 1 °C/min for (d).

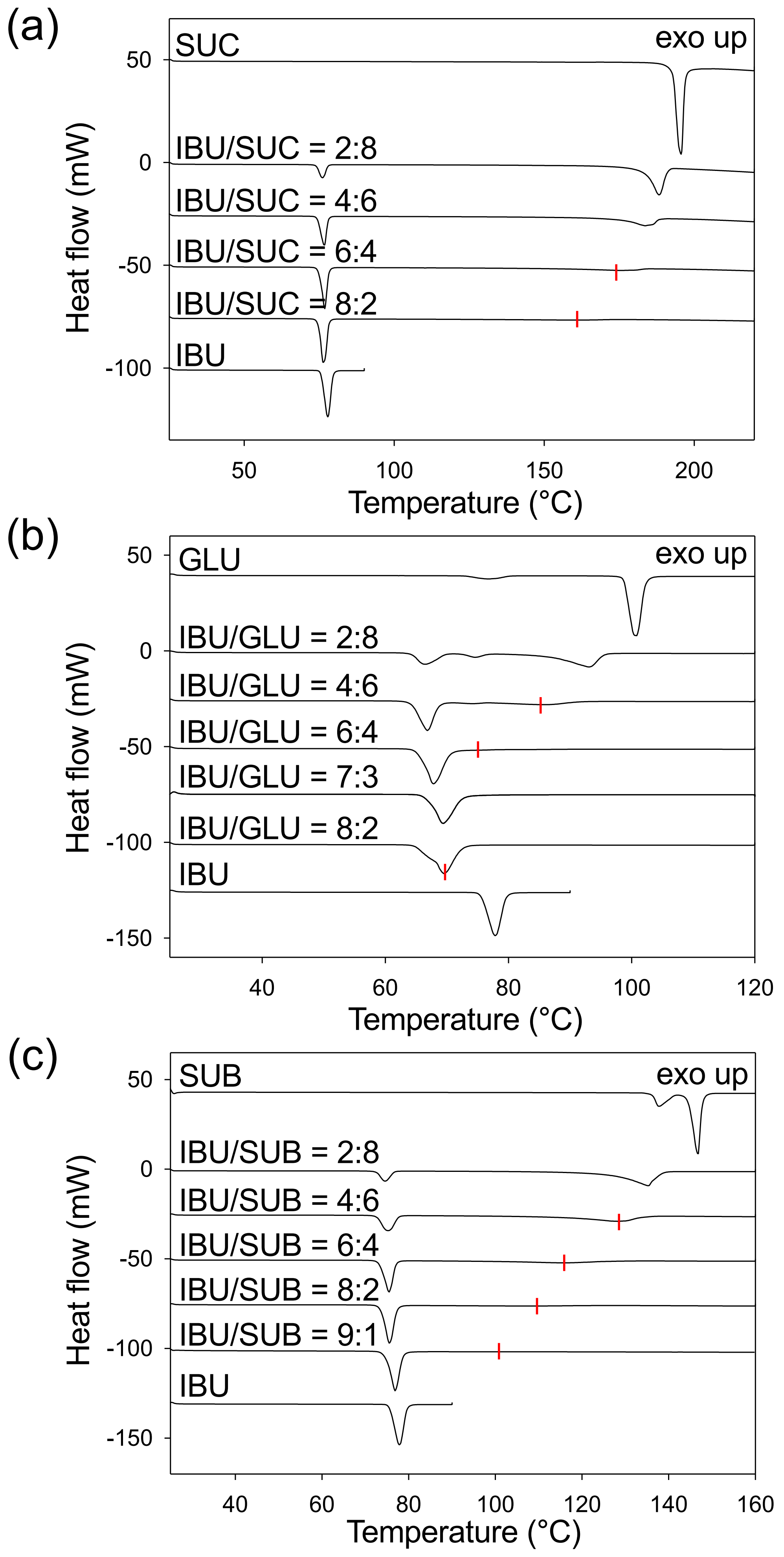

Figure S3. DSC thermograms of (a) IBU/SUC, (b) IBU/GLU, and (c) IBU/SUB mixtures. Heating rate 10 °C/min.

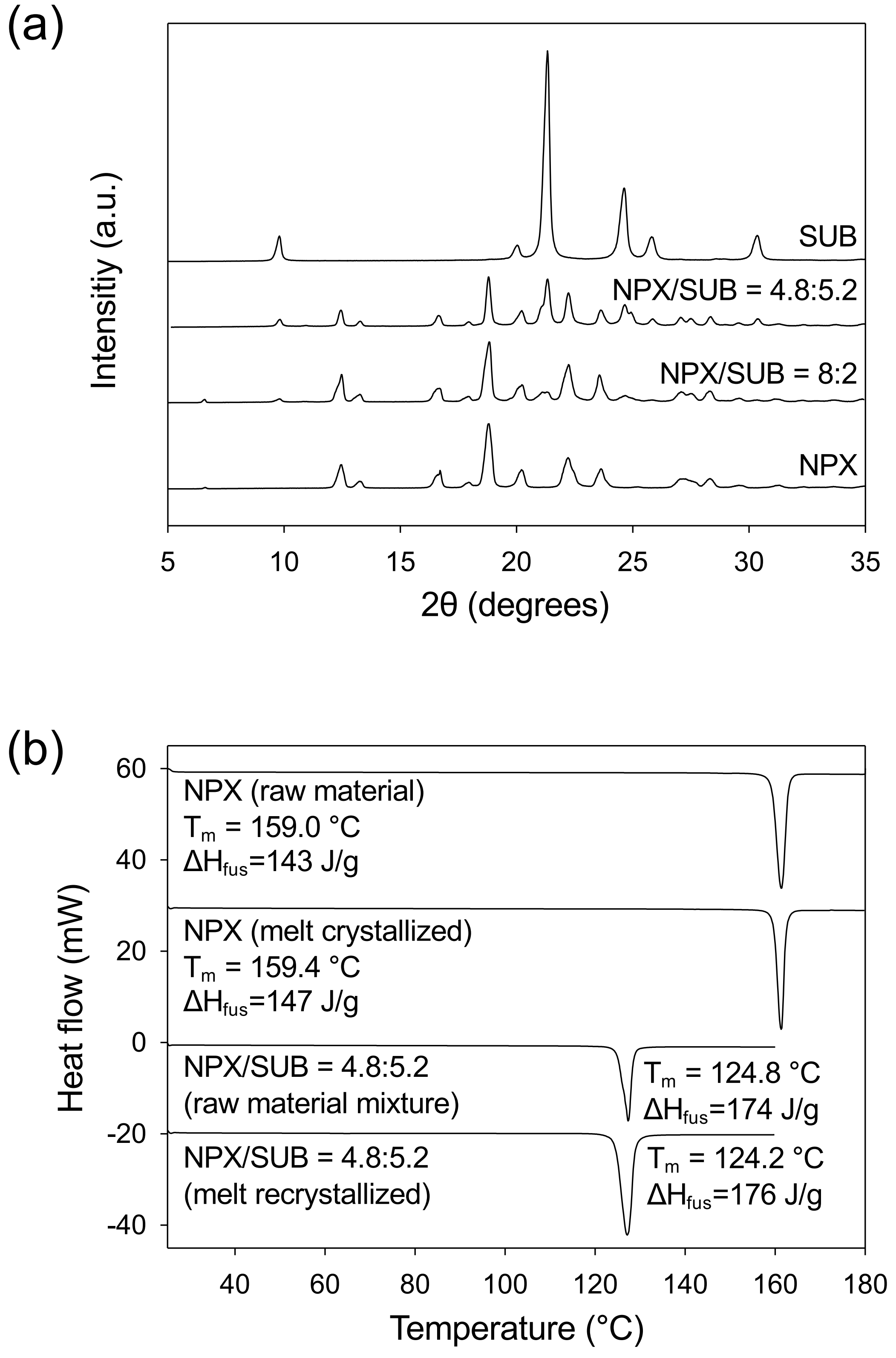

Figure S4. (a) XRD patterns for melt crystallized NPX, NPX/SUB, and SUB. (b) DSC thermograms to compare NPX and NPX/SUB before and after melt crystallization.

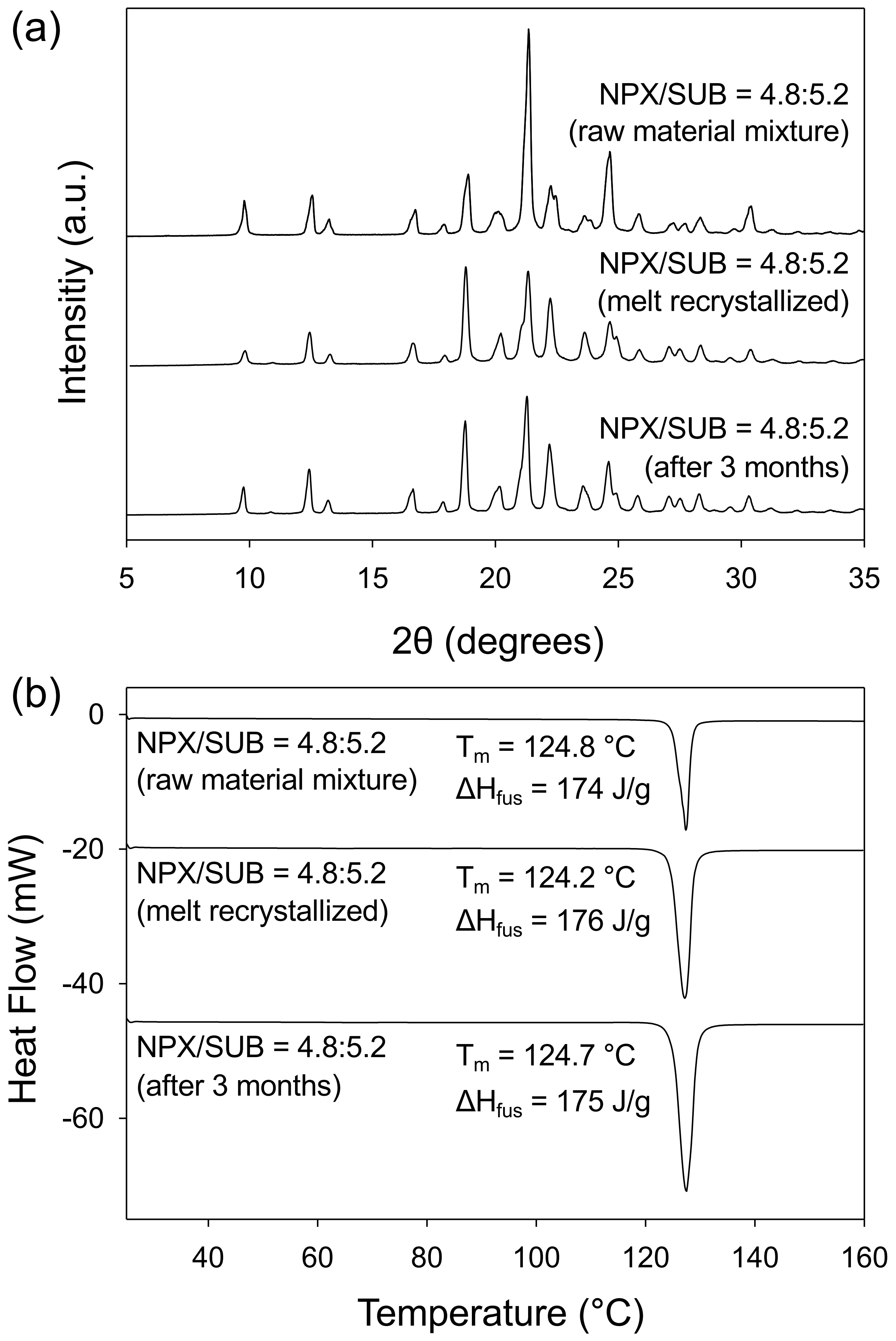

Figure S5. (a) XRD patterns and (b) DSC thermograms to show that virtually no change occurred for the 3-month storage (room temperature) after melt recrystallization.

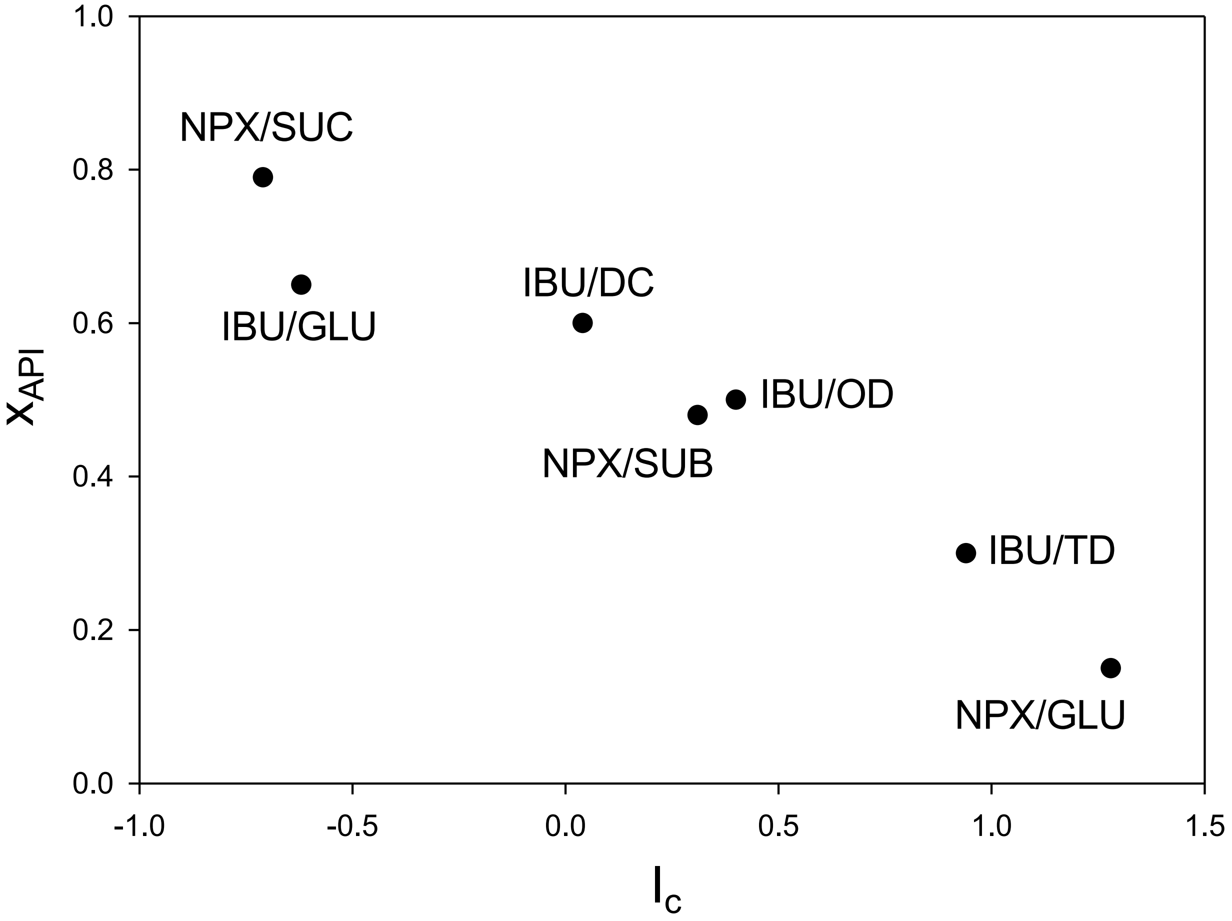

Figure S6. Correlation between I_c and the mole fraction of APIs (x_{API}) at eutectic points. The linear regression generated $x_{API} = -0.2832\ I_c + 0.5621$ ($R^2 = 0.9429$).
